# Protective Effects of *Ginkgo biloba* Extract EGb 761 against Noise Trauma-Induced Hearing Loss and Tinnitus Development

**DOI:** 10.1155/2014/427298

**Published:** 2014-06-17

**Authors:** Konstantin Tziridis, Sabine Korn, Sönke Ahlf, Holger Schulze

**Affiliations:** Experimental Otolaryngology, University of Erlangen-Nuremberg, Waldstraße 1, 91054 Erlangen, Germany

## Abstract

Noise-induced hearing loss (NIHL) and resulting comorbidities like subjective tinnitus are common diseases in modern societies. A substance shown to be effective against NIHL in an animal model is the *Ginkgo biloba* extract EGb 761. Further effects of the extract on the cellular and systemic levels of the nervous system make it a promising candidate not only for protection against NIHL but also for its secondary comorbidities like tinnitus. Following an earlier study we here tested the potential effectiveness of prophylactic EGb 761 treatment against NIHL and tinnitus development in the *Mongolian gerbil*. We monitored the effects of EGb 761 and noise trauma-induced changes on signal processing within the auditory system by means of behavioral and electrophysiological approaches. We found significantly reduced NIHL and tinnitus development upon EGb 761 application, compared to vehicle treated animals. These protective effects of EGb 761 were correlated with changes in auditory processing, both at peripheral and central levels. We propose a model with two main effects of EGb 761 on auditory processing, first, an increase of auditory brainstem activity leading to an increased thalamic input to the primary auditory cortex (AI) and second, an asymmetric effect on lateral inhibition in AI.

## 1. Introduction

A universal characteristic of modern societies, both in developing and developed countries, is the steadily increasing level of noise exposure within our working environments and during leisure time activities (for review see [1]). Consequently, an increasing number of people suffer from hearing disorders that result from an overexposure to noise, that is, noise-induced hearing loss (NIHL). Statistical data show that in 2000 about 9% of the general population in the USA display hearing impairments [2]. Similar data were published for Germany in 2006, where 8% of the 18- to 79-year-old adults appear to be hearing impaired [3]. Even more alarming are current data on the hearing loss in schoolchildren and teenagers. Several studies from North America and Europe report up to 15% of this age group to readily display significant hearing deficits [4], which reflects an increase of hearing loss among children and adolescents of around 10% [2, 3]. As the prevalence of hearing impairments increases with age, it appears to be sensible to assume that when these children grow up the number of patients with hearing impairments will increase dramatically in the future. Furthermore, as hearing loss may be etiologically responsible for the development of a number of secondary diseases, like hyperacusis (for review see [5]), tinnitus [6, 7], or depression due to social isolation [8], the problem of NIHL should be of immensely growing importance. For tinnitus alone, between 5% and 15% of the general population report to be affected and around 1% state that their quality of life is considerably impaired by their persistent perception of a phantom sound [9].

Effective strategies for protective measures against the development of hearing loss after noise exposure are therefore gaining increasing relevance in health care policies. In this context, two main types of strategies are conceivable: reducing noise exposure by technical measures or preventing the development of NIHL via pharmacological interventions. Whereas many technical measures, for example, for noise reduction in working environments or public, have the advantage that they may be effective for a large number of people, they are often very expensive and have no effect if the source of noise is self-inflicted [10].

During the last few years a large number of substances have been tested both in animal and human studies in search of a powerful drug that is able to prevent NIHL. Based on their physiological mechanisms of effectiveness against NIHL, a number of substance classes may be distinguished. Among these, antioxidants that reduce oxidative stress by elimination of reactive oxygen species (ROS) [11], glucocorticoids, and substances that improve cochlear blood flow (for review see [12]), activate inhibitory transmitter systems [13], or block apoptosis pathways in hair cells were most successfully employed (for review see [14]).

A substance that has been shown to protect against NIHL in guinea pigs is the* Ginkgo biloba* extract EGb 761 [15, 16]. Stange and coworkers demonstrated that animals which were treated with EGb 761 before exposing them to different types of noise trauma exhibited a smaller reduction in auditory nerve compound action potentials (CAP) than untreated controls. EGb 761 is a plant extract that is composed of about 80 different compounds which deploy not only one but also a number of different mechanisms presumed to counteract the development of NIHL. Well-documented are the protection of neuronal mitochondrial ATP synthesis in the presence of oxidative stress [17, 18], the protection of erythrocyte membranes against oxidative damage, which results in reduced blood viscosity and improved blood flow [19, 20], and neuroprotection through antiapoptotic properties [21–25]. In addition, the extract displays a number of effects that may counteract the development of secondary consequences of NIHL like tinnitus. These include increased extracellular dopamine levels in the prefrontal cortex [26], which may reduce depressive behavior that may foster the development of tinnitus [27], by partial inhibition of the norepinephrine transporter [28] or adult neurogenesis of hippocampal neurons [29], which additionally could both lead to cognition increasing effects. In clinical trials, the safety profile of the compound was similar to placebo [30]. Therefore, EGb 761 is a promising candidate substance for protective measures against NIHL and its consequences.

As detailed above, one of the consequences of NIHL may be the development of a tinnitus percept. In a previous study we have described the development of noise trauma-induced tinnitus in the* Mongolian gerbil* both on a behavioral and neurophysiological level [31]. We were able to detect a number of neuroplastic changes in auditory brainstem and cortex that were correlated with the development of a tinnitus percept as tested with a well-characterized behavioral paradigm [32]. In particular we could demonstrate a neuronal predisposition for the development of tinnitus. We were able to show that animals developing such a mispercept after an acoustic trauma show significantly less cortical activity already in the healthy state compared to tinnitus-resistant animals. The latter animals are able to counteract tinnitus development after noise trauma while animals without this ability develop a chronic tinnitus percept.

While studies in humans with different* Ginkgo biloba* extracts so far failed to show any reliable effect on tinnitus perception when given after its development [34, 35], we here tested the effectiveness of a prophylactic treatment with EGb 761 in the context of NIHL and tinnitus development in this animal model. We describe the effects of the EGb 761 extract on the behavioral level (acoustic startle response (ASR) audiometry), the auditory brainstem level (electrophysiological recordings of auditory brainstem responses (ABR)), and the central level (electrophysiological recording of local field potentials (LFP) and single and multiunit responses in auditory cortex (AC)). Our results point to massive neuroplastic effects of EGb 761 on auditory processing both at the peripheral and central level. These changes in processing may underlie the observed protective effects against NIHL and in the consequence tinnitus development.

## 2. Material and Methods

### 2.1. Ethics Statement and Animals

Mongolian gerbils (*Meriones unguiculatus*) were housed in standard animal racks (Bio A.S. Vent Light, Ehret Labor-und Pharmatechnik, Emmendingen, Germany) in groups of 2 to 3 animals per cage with free access to water and food at 20 to 24°C room temperature under 12/12 h dark/light cycle. The use and care of animals were approved by the state of Bavaria (Regierungspräsidium Mittelfranken, Ansbach, Germany).

A total of 36 ten- to twelve-week-old male gerbils purchased from Charles River Laboratories Inc. (Sulzfeld, Germany) were used in this study. All methods used in this paper have been described previously (Ginkgo treatment [36]; tinnitus model, behavioral audiometry, and electrophysiology [31]) but still will be recapitulated here for easier intelligibility.

### 2.2. Treatment with EGb 761 and Time Regime

EGb 761 is a dry extract from* Ginkgo biloba* leaves (35–67 : 1), extraction solvent: acetone 60% (w/w). The extract is adjusted to 22.0%–27.0% ginkgo flavonoids calculated as ginkgo flavone glycosides and 5.0%–7.0% terpene lactones consisting of 2.85%–3.4% ginkgolides A, B, and C and 2.6%–3.2% bilobalide and contains less than 5 ppm ginkgolic acids.

**Figure 1:**
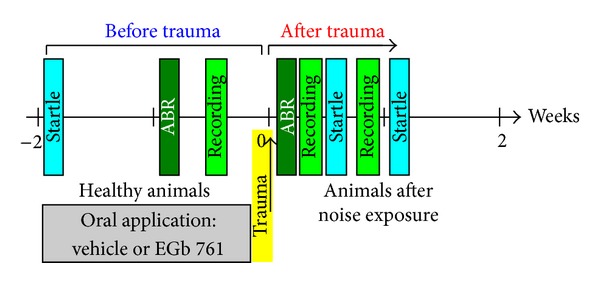
Timeline of the experiments. Two weeks prior to trauma (yellow bar) oral application of vehicle or EGb 761 was performed on a daily basis. Pretrauma measurements included behavioral startle responses (turquoise; hearing threshold and gap-noise tinnitus paradigms), ABR measurements (dark green), and electrophysiological recordings in auditory cortex (light green) both under anesthesia. After the acoustic trauma the measurements were repeated within the first seven days after the trauma.

EGb 761 provided by Dr. Willmar Schwabe Pharmaceutics (Karlsruhe, Germany) was diluted in 2% agar in water. As illustrated in Figure 1 the animals were either fed daily with the extract in agar (100 mg extract/kg body weight) via a feeding cannula over two weeks before start of the experiments (EGb 761 ginkgo group E, 17 animals), or they were fed over the same time with the same volume of agar only (vehicle control group V, 19 animals).

During the oral administration the measurements were started (cf. below and Figure 1). These included the behavioral testing in the first week of application, the pretrauma auditory brainstem response (ABR) measurements, and the pretrauma recording from the AC within the second week of substance administration. Subsequently, an acoustic trauma at 2 kHz was inflicted and all postmeasurements were done within 7 to 8 days after trauma.

### 2.3. Behavioral Measurements

For behavioral testing, animals were placed into a transparent acrylic tube (length: 10 cm; inner diameter 4.3 cm). This tube was placed 10 cm from a speaker (Canton Plus X Series 2) onto a Honeywell FSG15N1A piezo force sensor (sensitivity 0.24 mV/gram; null shift at ±25°C is ±1 mV; force range 0 to 1500 gram), assembled within an IAC acoustic chamber on a TMC low-vibration table. The front end of the tube was closed with a stainless steel grate (wire mesh width 0.5 mm) allowing acoustic stimulation with no detectable distortion (signal to noise ratio at least 70 dB). Sound pressure level was controlled via a B&K Type 2610 measuring amplifier fed with a B&K Type 2669 preamplifier/B&K Type 4190 condenser microphone combination. Stimulus generation and data acquisition were controlled using custom-made Matlab 2008 programs (MathWorks, Natick, MA, USA; stimulation/recording sampling rate 20 kHz). For sound generation the frequency response function of the speaker was calibrated to produce an output spectrum that was flat within ±1 dB.

**Figure 2:**
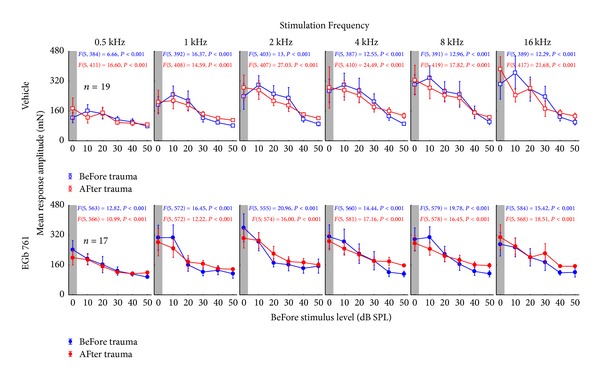
Validation of the hearing threshold ASR paradigm. Given are the mean response amplitudes in mN (±95% confidence interval) for all vehicle treated animals (upper panels) and all EGb 761 treated animals (lower panels) over all prestimulus intensities in dB SPL for the different stimulation frequencies of pre- and startle stimuli. The “pretrauma” (blue) and “posttrauma” (red) data are presented with the corresponding *F* statistics of the 1-factorial ANOVAs. Note that all statistics were significant, demonstrating that the animals were always (pre- and posttrauma = trauma status) responding to the 90 dB SPL startle stimulus and the different prestimuli.

**Figure 3:**
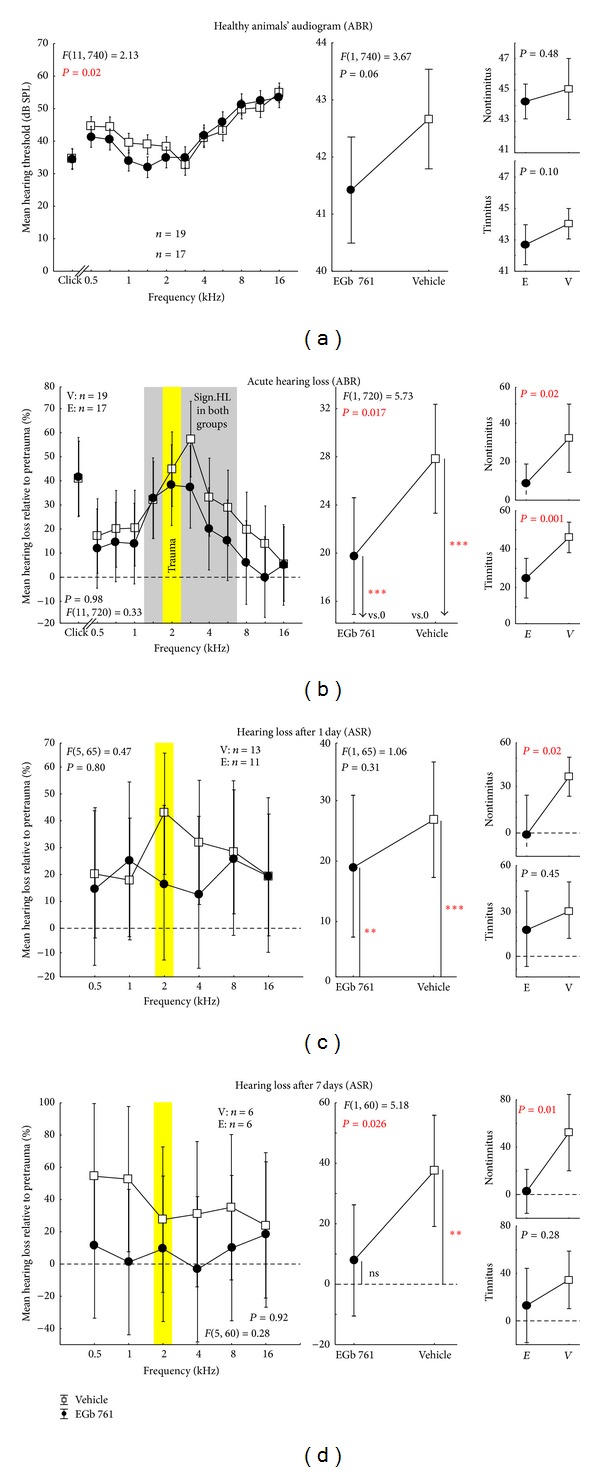
Hearing threshold and NIHL of all tested animals. (a) Auditory brainstem response (ABR) based audiogram of the healthy animals (before acoustic trauma) of vehicle group (black open squares) and EGb 761 treated group (black solid circles). The left panel documents the mean hearing thresholds with their 95% confidence interval for clicks and all tested tone frequencies with the *F*-statistics of the interaction of the 2-factorial ANOVA. The center panel depicts the 1-factorial part of the same ANOVA with the factor group (mean values over all tested frequencies and click). Right panels show the same data separated into animals that do not develop a tinnitus percept (upper panel) and those that did show a tinnitus percept after the trauma (lower panel). (b) Acute NIHL, relative to pretrauma in percent (change of ABR threshold relative to pretrauma) of both groups obtained by ABR, measured immediately after trauma at 2 kHz (yellow bar) with their 95% confidence interval. The grey area in the left panel indicates significant hearing loss (single sample *t*-test versus 0) in both groups (V = vehicle, E = EGb  761), which is also significant if averaged over all tested frequencies and the click stimulus (center panel, asterisks). (c) Hearing loss one day after trauma and (d) 7 days after trauma obtained by auditory startle response audiometry (see Section 2 for details). Note that relative changes of thresholds measured with either ABR or ASR have been demonstrated to be identical [33]. Symbols and abbreviations as above, single sample *t*-test: ns = not  significant, **P < 0.01, ***P < 0.001.

Three different types of prepulse inhibition (PPI) modulated auditory startle response (ASR) paradigms [31] were performed to assess, first, hearing capacities (behavioral audiogram, [37]) and, second, the potential existence of a tinnitus percept [38] after the noise trauma (cf. below). For obtaining the behavioral hearing thresholds we used a PPI of ASR paradigm in all animals. We startled the animals with 90 dB SPL pure tones (6 ms length including 2 ms rise and fall ramps) ranging from 0.5 kHz to 16 kHz in octave steps and used the same pure tones as prestimulus probes ranging from 0 to 50 dB SPL in 10 dB steps 100 ms before the startle stimulus. Each pure tone frequency and prestimulus intensity were repeated 15 times. This procedure was performed before the acoustic trauma and during the week after that event. The data obtained were checked by eye via a custom-made Matlab program; trials in which the animals moved within 100 ms before the startle stimulus were discarded; in the valid trials only peak-to-peak amplitudes of responses within the first 50 ms after startle stimulus onset were used for further analysis. The evaluation was performed independently by the principal investigator and a technical assistant, who was blind to the state of the animal. Evaluations of both experimenters led to identical results. This reduction of data led to a final valid trial number of 12260 of 20520 (59.7%) in the V group and 10719 of 18360 (58.4%) in the E animals. We made sure that the animals were always (pre- and posttrauma = trauma status) responding to the 90 dB SPL startle stimulus (cf.  Figure 2). For validation of the PPI effect of the prestimuli we performed 1-factiorial ANOVAs for the valid response amplitudes dependent on the prestimulus intensity for each frequency and trauma status separately for each individual animal. The mean responses of all 36 animals (19 V, 17 E) before and after trauma are given in Figure 2. Responses in this threshold paradigm were fitted with a sigmoidal Boltzmann functions for each frequency, trauma status, and animal separately. Hearing thresholds were defined as the sound level at the inflection point of the Boltzmann function at each frequency before and after trauma [33] and are depicted in Figure 3.

**Figure 4:**
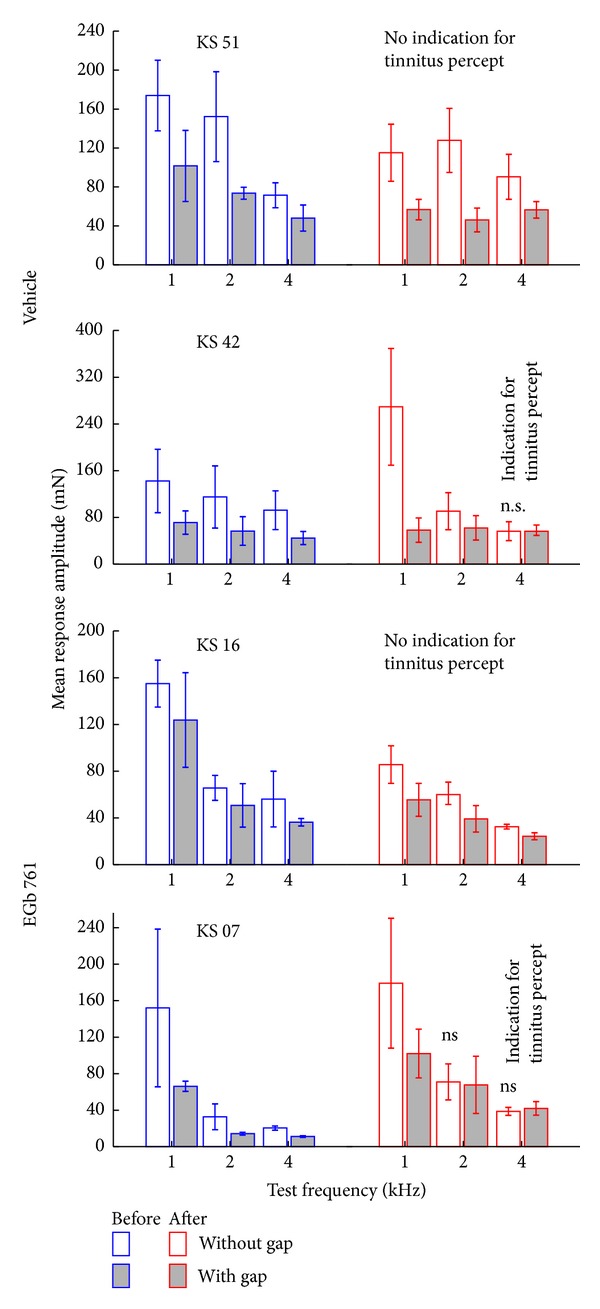
Results of the gap-noise PPI of the ASR in four exemplary animals. Given are the mean response amplitudes in mN (±standard deviation) for the two noise conditions: without gap (open bars) and with gap (filled bars) before (blue) and after (red) the trauma for all 3 frequencies tested (averaged for both gap noise paradigms). The upper two animals received the vehicle and the lower two animals received the EGb 761 extract before the trauma. All gap conditions produced significantly (*t*-tests) lower ASR amplitudes before the trauma. In some animals (KS 51 and KS 16) this was also true for the “posttrauma” condition and was a first indication for NT categorization. In other cases (KS 42 and KS 07) gap detection was impaired and did not show any significant change after the trauma at least at some frequencies; this was a first indication of T categorization (cf.  Section 2).

**Figure 5:**
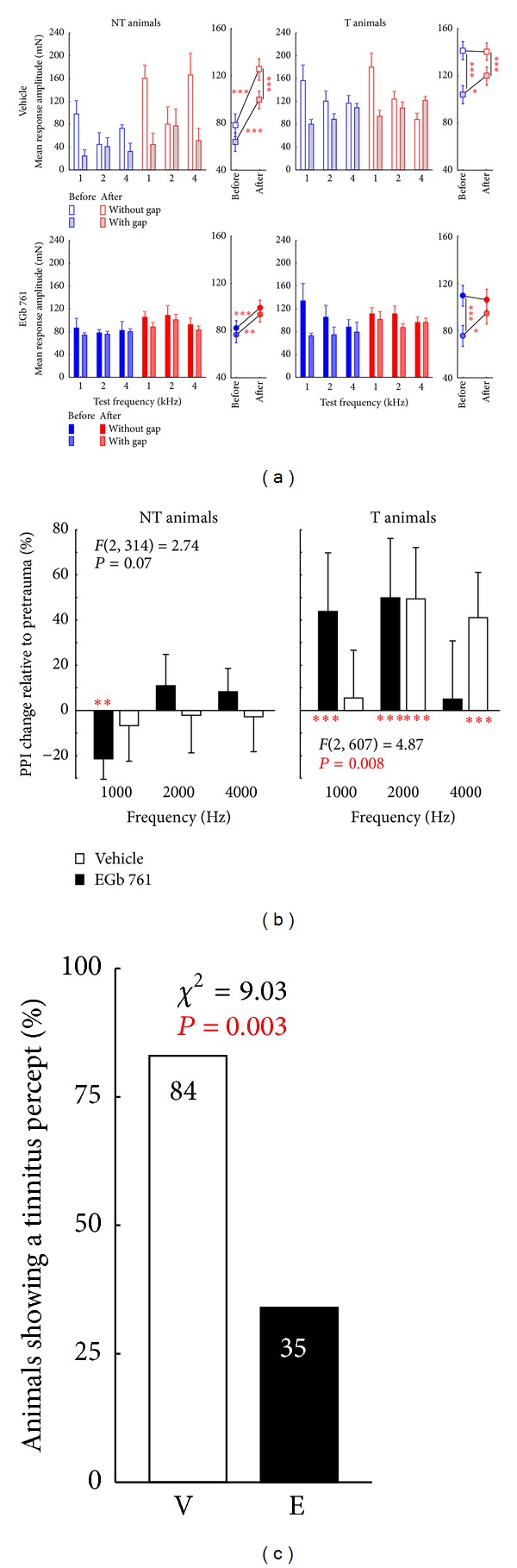
Development of tinnitus percept after acoustic trauma at 2 kHz. (a) Mean startle amplitudes in mN (+95% confidence interval) for no-gap (open and solid symbols) and gap condition (gray and shaded filled symbols) of all animals separated by tinnitus development, treatment, and test frequency. 2-factorial ANOVA (only interaction shown) depict the changes in no-gap and gap conditions before and after trauma. Note that even when gap-effects are small on the group level they were always significant in the single animals before trauma. Asterisks indicate significance levels of post hoc Tukey tests: *P < 0.05, **P < 0.01, ***P < 0.001. (b) Change of PPI relative to pretrauma data. Significant positive values of PPI change reflect an impaired PPI, indicating the development of a tinnitus percept. 2-factorial ANOVA indicates that EGb 761 treated animals develop tinnitus percepts at lower frequencies than vehicle treated controls. Asterisks below or above the abscissa indicate significant change of PPI (*t*-test versus 0): **P < 0.01, ***P < 0.001. (c) Percentage of animals that develop a tinnitus percept after an acoustic trauma at 2 kHz. EGb 761 treated animals show significantly less signs of a tinnitus percept (chi^2^ test).

For tinnitus testing we used two modified ASR paradigms in all animals before and after trauma. These consisted of either a 90 dB SPL pure tone startle stimulus of 1 kHz, 2 kHz, or 4 kHz within a 50 dB SPL continuous white noise, or a 90 dB SPL click startle pulse within a 50 dB SPL band pass filtered noise with a center frequency of 1 kHz, 2 kHz, or 4 kHz and a band width of one octave (cf. [31]). In both cases, a silent 20 ms gap within the noise 100 ms before the startle stimulus served as prepulse. The rational of these paradigms is that an animal that perceives tinnitus would be impaired in gap detection because it would hear its own tinnitus within the silent gap (cf. [32]). Consequently, when using a gap as prepulse, animals with tinnitus should produce smaller PPI of ASR than animals without tinnitus (cf.  Figure 4). If tinnitus is detected, the different tone frequencies and noise spectra used should give a rough estimate of the spectral content of the tinnitus percept. Each frequency and gap-condition were repeated 15 times and each test was performed before and after the acoustic trauma. Again the data obtained were checked by eye by the same two experimenters as above via a custom-made Matlab program. Trials in which the animals moved within 100 ms before the startle stimulus were discarded; in the valid trials only peak-to-peak amplitudes of responses within the first 50 ms after startle stimulus onset were used for further analysis [31]. This reduction of data led to a final valid trial number of 4582 of 6840 (67.0%) in the V group and 3630 of 6120 (59.3%) in the E animals. This approach allowed the determination of a possible frequency-specific tinnitus-related behavior at one octave precision. We tested the gap-effect on the response amplitude separately in each individual animal before and after trauma for each tested frequency by *t*-tests (*α* = 0.05) and found in all pretrauma data a significant PPI effect (i.e., a reduction of startle amplitude in the condition with the gap in the background noise) in each individual animal (*P* < 0.05). After the trauma only a part of the animals showed an undisturbed gap-effect at all frequencies tested while other animals showed no gap-effect at some but not all frequencies (cf.  Figure 4) which gave a first hint of a possible tinnitus percept but was not yet used as the final classification of the animals in the tinnitus or nontinnitus group (cf. below). To avoid possible effects of the acoustic trauma on different stimulation frequencies all startle response data were normalized to minimize variance of the response amplitudes. Normalization was performed as described earlier [31, 39]; briefly, we divided each amplitude by the corresponding median amplitude of the 90 dB SPL only condition (which reflects the full startle response of the animal for the loudest condition at each specific frequency, grey area in Figure 2). Thus we were able to control for differences in the startle amplitudes resulting from the hearing loss at the trauma frequency. This normalization also guarantees that the reduced ASR response after acoustic trauma is not due to hearing loss rather than a tinnitus percept [39]. Finally, the PPI of ASR in the healthy animal (before trauma) and after the trauma was calculated and the change in PPI relative to pretrauma (in %) was tested against 0 (no change) for each frequency separately with a *t*-test (*α* = 0.025). Significant positive values for PPI change reflect impaired PPI and therefore indicate the development of a tinnitus percept. Only such animals were therefore classified as probably perceiving tinnitus (T groups) that showed at least one impaired frequency after the trauma independent of the affected frequency itself. As it turned out, in all cases where tinnitus was detected according to one of the two gap-ASR paradigms used, the second gap-ASR paradigm was also positive for tinnitus. Only the affected frequencies could differ between gap-ASR paradigms. Animals without such a significant increase in PPI change were classified as nontinnitus perceiving animals (NT groups) (cf.  Figure 5). As it turned out, animals classified as T or NT based on these behavioral measures also differed in neurophysiological response measures (ABR and AC; for example, Figures 7 and 12), thereby strengthening the classification (cf. also [40]).

### 2.4. Acoustic Trauma and Auditory Brainstem Recordings (ABR)

A bilateral acoustic trauma at 2 kHz (Canton Plus X Series 2 speaker frontal at 10 cm distance from animals head, 115 dB SPL at animals head, 75 min duration) in deep ketamine xylazine anesthesia (mixture of ketamine, xylazine, NaCl, atropine at a mixing ratio of 9 : 1 : 8 : 2, initial dose: 0.3 mL s.c.; continuing application at a rate of 0.2 to 0.3 mL/h) was used to induce a frequency-specific NIHL in all 36 animals and possibly the subsequent development of a tinnitus percept. The animals' body temperature was kept constant at 37°C by a warming pad.

ABRs were measured via subcutaneously placed thin silver wire electrodes (0.25 mm diameter) using a Plexon Multichannel Acquisition Processor (HLK2, Plexon Inc., Dallas, TX, USA) after amplification by a JHM NeuroAmp 401 (bandpass filter 400 Hz to 2000 Hz, 50 Hz notch filter) and stored with a custom-made Matlab program (10 kHz sampling rate). Auditory stimuli were presented free field to one ear at a time via a frequency response function corrected speaker (SinusLive neo 25S, pro hifi, Kaltenkirchen, Germany) at circa 0.5 cm distance from the animal's pinna while the contralateral ear was tamped with an ear plug as previously described [41]. Stimuli presented were clicks (0.1 ms duration) and pure tones (4 ms duration including 1 ms cosine-squared rise and fall times) ranging from 0.5 to 16.0 kHz in half-octave steps. 120 stimuli were presented in pairs of two-phase inverted stimuli (intrastimulus interval 100 ms) and an interstimulus interval of 500 ms between stimulus pairs. Stimulation was pseudo-randomized using a fixed list of all combinations of stimulus frequencies and sound pressure levels (0 to 90 dB SPL in 5 dB steps). To obtain ABR-based audiograms the mean ABR waves were compared to the mean amplitude 200 to 100 ms before the stimulus (baseline). Thresholds were defined automatically by a custom-made Matlab program at the highest attenuation at which the evoked amplitude raised over 2 standard deviations of the baseline; data were discarded at frequencies where this procedure was not possible, for example, at low signal to noise ratios. For additional analysis the root mean square (RMS) value of the ABR signal was calculated from 1 to 5 ms after stimulus onset. For further analysis data from both ears of each animal were used.

As behavioral audiograms using PPI of ASR (cf. above) could be obtained much faster—although with lower frequency resolution—than the ABR recordings (1.5 h compared to 6 h) we decided to measure fine-grain audiograms before and immediately after noise trauma only. For later audiogram measurements we rely on behavioral audiograms only (cf. Figure 1). We have demonstrated earlier [33] that these different methods to assess audiograms in our animal model yield different absolute thresholds but identical relative shifts in hearing thresholds after noise trauma. As we compare only relative shifts in this study (cf. Figure 3) this is not expected to introduce any bias to the interpretation of our findings presented here.

### 2.5. Electrophysiological Unit Recordings in Primary Auditory Cortex (AI)

In a subset of the 36 animals (3 vehicle, 4 EGb 761) used in this study we performed electrophysiological recordings in auditory cortex in addition to the behavioral and ABR measurements described above. Two to three days after obtaining baseline ASR and ABR data, that is, before the acoustic trauma, the skull of the anesthetized animals was trepanned to expose the left auditory cortex. A 2.5 cm aluminum head-post for fixation and a recording chamber were implanted. Recording under deep ketamine-xylazine anesthesia began two days after surgery. Single and multiunit responses to tones were recorded in primary auditory cortex (AI) using acutely inserted single tungsten microelectrodes (1 MΩ impedance, 1-2 *μ*m tip diameter, Plexon microelectrodes PLX-ME-W-3-PC-3-1.0-A-254). Verification of recording sites was done using neuronal response characteristics (latency, tuning sharpness (*Q*_30_), temporal response patterns (phasic/tonic), tonotopic organization [42]). We concentrated our investigation on units with phasic response patterns.

Stimulation consisted of pure tones (200 ms including 1 ms cosine-squared rise and fall times) ranging from 0.25 to 16.0 kHz in quarter-octave or half-octave steps presented pseudo-randomly at 70 dB SPL with 500 ms interstimulus intervals. In addition to these iso-intensity measurements, tuning curves were recorded using pure tones in the mentioned frequency range but at different intensities ranging from 0 to 90 dB SPL. The recorded unit activity was analyzed with custom-made Matlab and IDL programs (IDL 7.06, Exelis Visual Information Solutions, McLean, VA, USA). Best frequency (BF; frequency with highest discharge rate at 70 dB SPL) as well as spontaneous rate (mean activity within a time window from 50 ms before to stimulus onset), evoked rate at BF, and evoked rates at all tested stimulation intensities and frequencies were calculated for each unit individually (evoked rate was calculated as the mean firing rate in a time window comprising the onset response, usually ranging from stimulus onset to 60 ms after stimulus onset). Statistics were performed with Statistica 8 (StatSoft, Hamburg, Germany). Where appropriate, either parametric statistics (Student's *t*-test, one- and two-factorial ANOVA with Tukey post-hoc-test) or nonparametric statistics (Kolmogorov-Smirnov-test, Kruskal-Wallis ANOVA with post hoc Median-test, and Mann-Whitney *U*-test) were applied.

## 3. Results and Discussion

### 3.1. Effects of Prophylactic EGb 761 Treatment on NIHL and Tinnitus Emergence

We used the PPI of the ASR to obtain behavioral audiograms from all 36 animals before and after the acoustic trauma. The responses to the different prestimulus intensities were checked by 1-factorial ANOVAs for each frequency and trauma status (before or after trauma) and for each individual animal separately. For an overview of the different response characteristics before and after trauma the mean response amplitudes of the 19 vehicle treated and 17 EGb 761 treated animals are given in Figure 2. Note that we find different response characteristics dependent on the stimulation frequency and trauma status but always find the significant prestimulus intensity effect of the PPI, that is, a decrease in ASR amplitude with increasing prestimulus intensity, which is not only true for the mean response but also for the individual responses. To these individual responses we fitted sigmoidal Boltzmann functions obtaining the individual behavioral hearing thresholds for each frequency.

Additional to these behavioral thresholds we obtained individual ABR based hearing thresholds under anesthesia before and after the trauma. Interestingly, when comparing the ABR thresholds of EGb 761 treated and vehicle treated animals, treatment led to slightly improved hearing thresholds in the low frequency range between 0.5 and 1.4 kHz already before induction of NIHL (Figure 3(a) left, filled circles and open squares, resp.), while across all frequencies, only a tendency for better hearing (Figure 3(a) center) could be found. Between the treated animals that later (cf. below) developed a tinnitus percept (T animals) and those that did not (NT animals) there was no significant difference in overall hearing level (Figure 3(a) right).

The traumatizing pure tone at 2 kHz led to significant and frequency specific NIHL immediately after the traumatizing event in both the EGb 761 treated group (E) and the vehicle treated control group (V) (Figure 3(b)). In both groups, significant elevations of hearing thresholds could be detected for frequencies between 1.4 and 5.6 kHz, indicating a stronger effect on the high frequency range compared to the low frequency range relative to the traumatizing pure tone (gray area in Figure 3(b) left). Nevertheless, the impact of the trauma on the hearing thresholds was significantly stronger in group V compared to group E (Figure 3(b) center). Whereas the mean threshold increase across all frequencies in group V was 27.8% relative to “pretrauma” (=9.0 dB), it was only 19.8% (=4.7 dB) in group E (Figure 3(b) center). There was no difference between NT and T animals in the relative NIHL (Figure 3(b) right). Furthermore, the degree of NIHL that developed differed between the V and E groups during the first week after trauma (Figures 3(c) and 3(d)). Whereas the frequency specificity of the NIHL vanished in both groups resulting in flat NIHL functions, the overall threshold shift completely recovered in group E, resulting in audiograms not significantly different from pretrauma conditions 7 days after trauma. In contrast, the NIHL in group V increased within the same time period (Figure 3(d)). The temporal development was entirely different between NT and T animals (Figures 3(c) right, 3(d) right). While NT animals showed no remaining hearing loss only 1 day after trauma, all vehicle treated animals and the T animals of the E group showed increased NIHL until day 7 after trauma. Note that we show the relative hearing loss compared to pretrauma level as we want to keep the two different methods of hearing threshold level measurements comparable. As we have demonstrated in an earlier study [33] ABR and ASR thresholds differ in absolute values so that audiograms show a parallel upward or downward shift. Relative changes due to hearing loss on the other hand were identical for both methods.

As described above, both EGb 761 and vehicle treated groups contained T and NT animals. Exemplarily the mean response amplitudes to both gap-ASR paradigms of four animals are depicted in Figure 4. The two upper animals were treated with the vehicle and the two lower ones with the substance. In animals KS 51 and KS 16 significant gap detection (*t*-tests, *P* < 0.05) was found before and after the trauma at all frequencies tested. In animals KS 42 and KS 07 that was only the case before the trauma, after the acoustic trauma gap detection was impaired at 4 kHz or 2 kHz and 4 kHz, respectively. This impairment was a first hint for the classification of these two animals into the tinnitus group (cf.  Section 2). The mean startle response amplitudes for all these animals before and after the acoustic trauma are depicted in Figure 5(a). Four 2-factorial ANOVA revealed especially in NT animals of both groups (Figure 5(a), left panels) significantly increased startle amplitudes after the trauma in the no-gap and in the gap condition (2-factorial ANOVAs; V group: trauma status: *F*(1, 324) = 90.73, *P* < 0.001; gap presence: *F*(1, 324) = 21.18, *P* < 0.001; interaction trauma status and gap presence: *F*(1, 324) = 1.75, *P* = 0.19; E group: trauma status: *F*(1, 788) = 27.34, *P* < 0.001; gap presence: *F*(1, 788) = 3.97, *P* = 0.04; interaction trauma status and gap presence: *F*(1, 788) = 0.001, *P* = 0.97) while in the T animals (Figure 5(a), right panels) only the gap conditions showed significantly elevated amplitudes (2-factorial ANOVAs; V group: trauma status: *F*(1, 1964) = 3.80, *P* = 0.051; gap presence: *F*(1, 1964) = 56.27, *P* < 0.001; interaction trauma status and gap presence: *F*(1, 1964) = 4.70, *P* = 0.03; E group: trauma status: *F*(1, 968) = 3.04, *P* = 0.08; gap presence: *F*(1, 968) = 25.26; interaction trauma status and gap presence: *F*(1, 968) = 6.14, *P* = 0.01). Please note that in this plot we show the means of the unnormalized response amplitudes of the animals. By that the gap-effect—especially in the “pretrauma” condition—is not always visible. As we used the individual data of each animal separately and calculated the PPI change relative to pretrauma the mean amplitudes give only a raw picture of the classification method (e.g., of individual data refer to Figure 4). So obviously, whereas the relative change in PPI after trauma relative to pretrauma conditions leads to stronger or nonsignificant PPI change in the NT animals (Figure 5(b), left panel), relative PPI amplitudes were significantly reduced in T animals (Figure 5(b), right panel; note that a positive relative PPI change in Figure 5(b) refers to a reduction in absolute posttrauma PPI amplitude). Consequently, in NT animals a two-factorial ANOVA showed no significant interaction of group (V versus E) and frequency in PPI change (Figure 5(b), left panel) and also no difference in the one-factorial part of the analysis (V versus E, *F*(1, 314) = 0.34, *P* = 0.56). As a result of the categorization of the individual data, no significant impairment of the PPI could be found. Interestingly, a significant decrease of PPI change emerged at 1 kHz in E group but not in V group animals (*t*-tests versus 0), indicating an improved PPI in this group. On the other hand, the PPI data of T animals from both groups showed significant interaction in the two-factorial ANOVA (Figure 5(b), right panel), indicating a spectrally different percept of animals in the E group compared to V group, namely, a tinnitus precept with lower frequencies. By contrast, across all frequencies we did not find any significant difference between both groups (*F*(1, 607) = 0.01, *P* = 0.93). It should be noted that, since EGb 761 treatment obviously provides considerable protection against NIHL, the impact of the tinnitus-inducing event affected the auditory system less severe in group E compared to group V. Consequently we found fewer animals in group E that developed a tinnitus percept compared to group V (cf.  Figure 5(c)). Whereas 84% (16/19) of animals in group V showed clear signs of tinnitus in our behavioral paradigms, significantly fewer (chi^2^ test, *P* = 0.003), namely, 35% (6/17) of the animals in group E seemed to have developed tinnitus.

### 3.2. Neurophysiological Effects of Prophylactic EGb 761 Treatment in AI

#### 3.2.1. Overall Neuronal Activity

**Figure 6:**
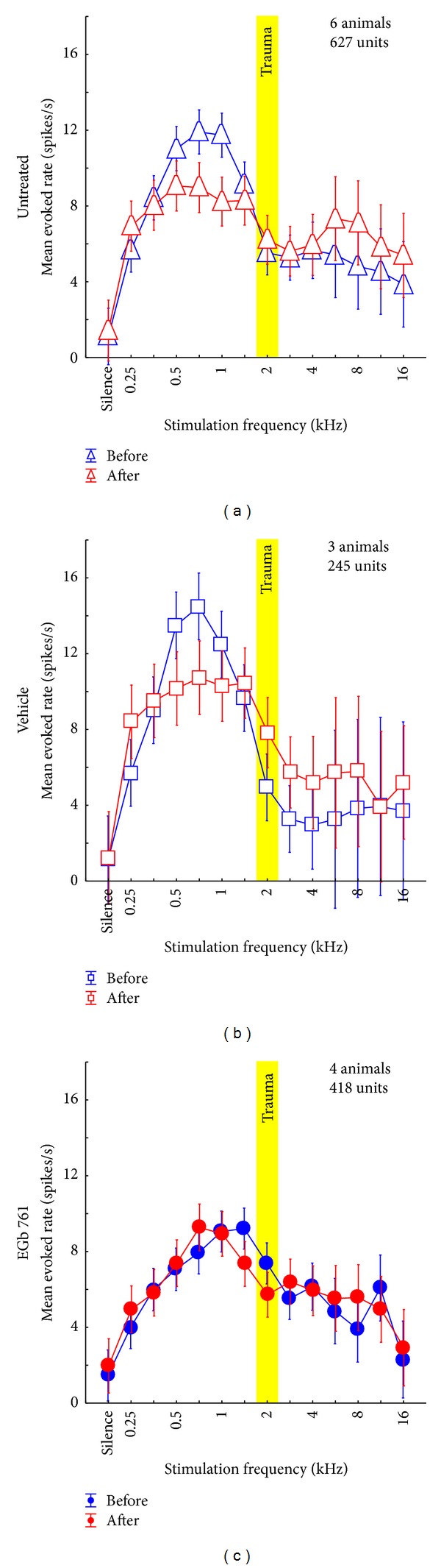
Mean evoked neuronal response (±95% confidence interval) to iso-intensity pure tone stimulation across all recorded units in (a) untreated animals from an earlier study [31], (b) vehicle treated animals, and (c) EGb 761-treated animals. Depicted are the mean evoked rates (spikes/s) before (blue) and after (red) acoustic trauma at 2 kHz (yellow bar). For statistical values please refer to Section 3.2.1.

In total, 663 units could be recorded in 7 of 36 treated animals (418 units in 4 E animals; 245 units in 3 V animals. Note that all statistics in this paragraph are based on unit numbers, not animal numbers). We first investigated the general effect of the application of the EGb 761 extract on cortical responses and compared it to the vehicle treated group and to an untreated group (U) of 6 animals from an earlier study (627 units) [31]. Neurophysiological responses to tones of single and multiunits in AI showed a number of significant differences between EGb 761 and vehicle treated animals (group E versus V), both before the induction of NIHL and in response to the noise trauma while group V showed nearly identical responses to the U animals (2-factorial ANOVA: group: *F*(2, 18480) = 27.83, *P* < 0.001; trauma status: *F*(1, 18480) = 1.05, *P* = 0.31; interaction: *F*(2, 18480) = 1.24, *P* = 0.29). Post hoc Tukey tests revealed a significant difference in mean responses before the trauma between U and E (*P* < 0.001) and V and E (*P* < 0.001) but not between U and V (*P* = 0.79) which was also true for the responses after the trauma (U versus E: *P* = 0.03; V versus E: *P* < 0.001; U versus V: *P* = 0.11).  Figure 6 gives an overview of the mean neuronal discharge activity in AI as a function of stimulation frequency and trauma status. We here compared pre- and posttrauma evoked responses across all stimulation frequencies by group with 2-factorial ANOVAs. Responses of the untreated group (Figure 6(a)) show no change in mean (± standard deviation) pre- and posttrauma response rates averaged across all frequencies (before: 7.57 ± 11.76 spk/sec; after: 7.00 ± 13.24 spk/sec; *F*(1, 7846) = 0.003, *P* = 0.95) but a frequency dependency (*F*(13, 7846) = 24.22, *P* < 0.001) while the interaction of both factors (*F*(13, 7846) = 2.86, *P* < 0.001) indicates a change of responses dependent on frequency and trauma status. Basically we see the same results in the vehicle treated group (Figure 6(b)) with no effect of the trauma status on mean response rate (before: 7.99 ± 10.91; after: 8.01 ± 16.00; *F*(1, 3938) = 1.19, *P* = 0.28) but a frequency dependency (*F*(13, 3938) = 20.27, *P* < 0.001) and the significant interaction of both factors (*F*(13, 3938) = 2.50, *P* = 0.002), demonstrating that handling and vehicle treatment per se had no effect on our measurements. The EGb 761 treated animals (Figure 6(c)) showed a somewhat dampened response when comparing it with the two other groups (cf. analysis above); the responses did not show an overall effect of the trauma (before: 6.28 ± 11.20 spk/sec; after: 6.26 ± 9.19 spk/sec; *F*(1, 6618) = 0.23, *P* = 0.63), although they did show a frequency dependency (*F*(13, 6618) = 18.63, *P* < 0.001) but no interaction (*F*(13, 6618) = 1.37, *P* = 0.17).

**Figure 7:**
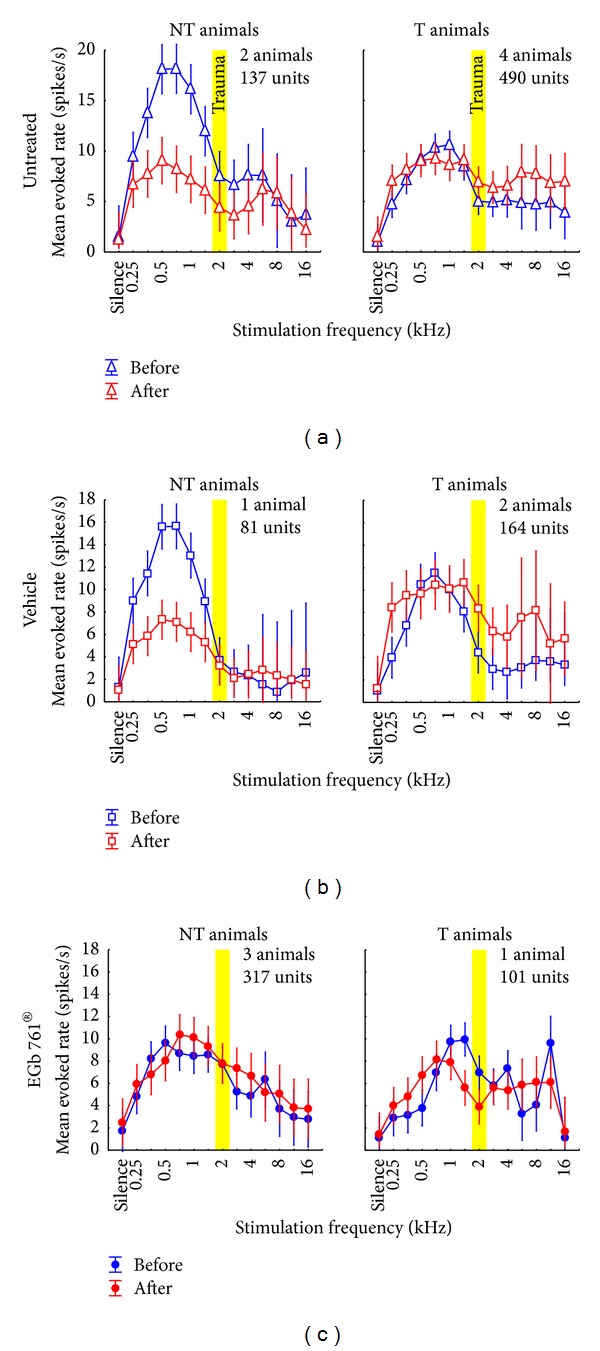
Mean evoked neuronal response (±95% confidence interval) to iso-intensity pure tone stimulation across all recorded units in non-tinnitus and tinnitus perceiving animals. Animals that did not develop a tinnitus percept are grouped in the left column while animals that perceived tinnitus are shown in the right column. Depicted are the mean evoked rates (spikes/s) before (blue) and after (red) acoustic trauma at 2 kHz (yellow bar). (a) Data from untreated animals replotted from an earlier study [31]. 2-factorial ANOVA interaction *F* statistics: NT: *F*(13, 1866) = 3.12, *P* < 0.001; T: *F*(13, 5952) = 1.54, *P* = 0.10. (b) Data from vehicle treated animals. 2-factorial ANOVA interaction *F*-statistics: NT: *F*(13, 838) = 8.46, *P* < 0.001; T: *F*(13, 2772) = 1.42, *P* = 0.14. Note the similarity between these and the untreated animals in the NT as well as in the T group. (c) Data from EGb 761 treated animals showing clear differences to the other two animal groups. The 2-factorial ANOVA shows strong interaction of time of measurement (before versus after trauma) and stimulation frequency in the T (*F*(13, 1970) = 5.58, *P* < 0.001), but not in the NT group (*F*(13, 4420) = 0.86, *P* = 0.59).

Of the 7 animals where single unit AC responses were recorded in this study, only one in the E group developed a tinnitus percept but two in the V group and therefore the following detailed analysis has a preliminary character, but as group V and group U show basically the identical response patterns in NT and T animals it still seems likely that we found a valid effect in our animal model.  Figure 7 gives an overview of the mean activity in AI as a function of stimulation frequency and trauma status. Mean response rates across all recorded units in completely untreated animals (Figure 7(a), data replotted from [31]), vehicle treated controls (Figure 7(b)), and EGb 761 treated animals (Figure 7(c)) are compared. Panels in the left column show data from animals that did not develop a tinnitus percept (NT); right panels depict data from those animals that did develop tinnitus (T) after NIHL as determined by the behavioral gap-noise paradigms.

The data of group V were very similar to our recently published results [31] with untreated animals (Figure 7(b) versus Figure 7(a)). In the vehicle treated animals we found a comparable overall activity in AI, both before and after trauma, for animals with and without tinnitus (mean responses averaged across all frequencies grouped by tinnitus status, *t*-tests always *P* > 0.05). Again, a predisposition for the development of tinnitus, obvious from an overall lower cortical activity before trauma, compared to the animal group that does not develop tinnitus after NIHL, could be demonstrated. Furthermore, the reduction of the initially high response rates in the low frequency range in the NT groups after trauma was similar in the vehicle treated and the untreated group and not seen in the T groups of animals that did develop a tinnitus percept (*t*-tests before versus after, low frequency range (mean (± standard deviation)): untreated NT: 14.6 (±15.9) spk/sec versus 7.5 (±8.7) spk/sec, *P* < 0.001; vehicle NT: 12.3 (±11.9) spk/sec versus 6.2 (±5.5) spk/sec, *P* < 0.001; untreated T: 8.4 (±11.7) spk/sec versus 8.5 (±16.5) spk/sec, *P* = 0.82; vehicle T: 10.4 (±12.3) spk/sec versus 12.0 (±21.8) spk/sec, *P* = 0.04). We hypothesize this high response rate to be a correlate of the mechanisms that prevents the development of tinnitus in these animals [31]. On the contrary, T group animals showed increased posttrauma response rates in the high frequency range above the trauma frequencies, corresponding to the behaviorally determined frequency range of their tinnitus percept [31] (*t*-tests before versus after, high frequency range: untreated NT: 3.9 (±6.9) spk/sec versus 3.9 (±7.9) spk/sec, *P* = 0.99; vehicle NT: 1.8 (±2.7) spk/sec versus 1.9 (±2.4) spk/sec, *P* = 0.82; untreated T: 4.5 (±7.8) spk/sec versus 7.2 (±16.2) spk/sec, *P* = 0.002; vehicle T: 4.2 (±4.1) spk/sec versus 7.4 (±8.7) spk/sec, *P* = 0.004). We conclude from this comparison of untreated and vehicle treated animals that the mere handling of animals that was associated with vehicle (or EGb 761) administration had no effect on overall activity in AI, neither “pre-” nor posttrauma.

**Figure 8:**
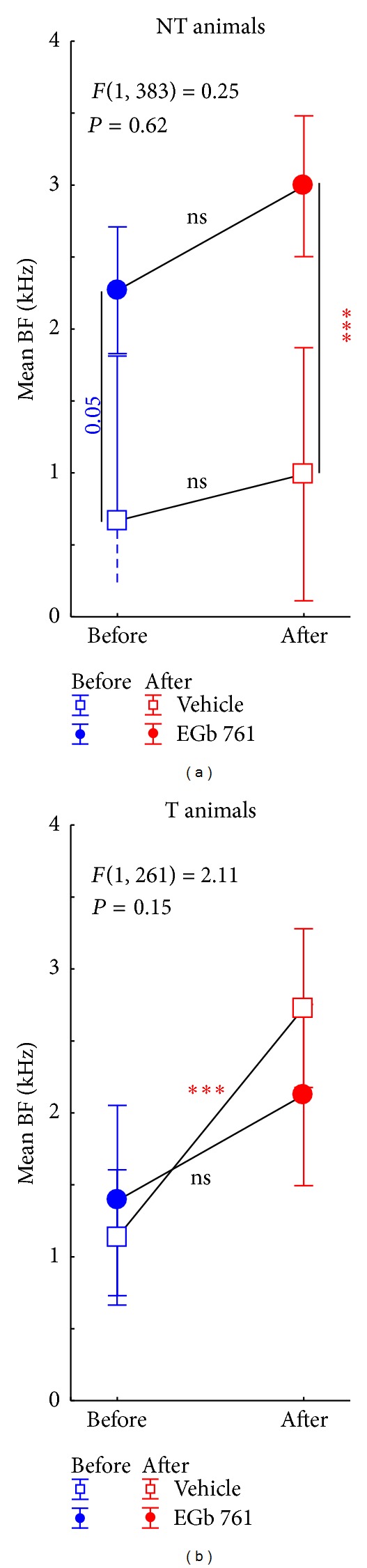
Effect of noise trauma on mean best frequency (BF) ±95% confidence interval in NT and T animals. Depicted are the statistical interactions of time of measurement (before versus after trauma) and animal group (V versus E) with the *F*-statistics of the 2-factorial ANOVAs. Asterisks indicate significant Tukey post-hoc-tests levels (ns = not  significant, ***P < 0.001). Note the offset between vehicle and EGb 761 treated animals in the nontinnitus animals' data (a).

In contrast to this high degree of similarity between data from untreated and vehicle treated animals, large differences were found in the overall activity of units in AI of the EGb 761 treated animals (group E, Figure 7(c)). In NT animals (Figure 7(c), left panel) we observed low overall activity similar to the activity seen in the group V-NT after trauma (Figure 7(b), left panel, red curve) even before inflicted trauma (Figure 7(c), left panel, blue curve). At the same time, the overall activity in AI as a function of stimulation frequency in group E-NT before trauma was also similar to that of group V-T before trauma (Figure 7(c), left panel, blue curve versus Figure 7(b), right panel, blue curve). However, in contrast to the latter, the EGb 761 treated group did not display increased response rates in the high frequency region after trauma (Figure 7(c), left panel, red curve versus Figure 7(b), right panel, red curve) and did not develop behavioral signs of tinnitus. Rather, the mean activity in AI in group E-NT showed no significant changes post trauma (2-factorial ANOVA: before versus after: *F*(1, 3420) = 2.67, *P* = 0.10; interaction: *F*(13, 3420) = 0.86, *P* = 0.59) and therefore seemed to be resistant against such NIHL induced plasticity. This stabilizing effect of EGb 761 on overall activity in AI seemed to be less effective in group E-T (2-factorial ANOVA: before versus after: *F*(1, 3170) = 0.24, *P* = 0.62; interaction: *F*(13, 3170) = 3.58, *P* < 0.001; cf. also Figures 8 and 10(b)), resulting in more noisy frequency response functions compared to group E-NT (Figure 7(c), right panel), which may be the reason why animals in this group V could not withstand the development of NIHL induced tinnitus.

#### 3.2.2. Spectral Tuning

**Figure 9:**
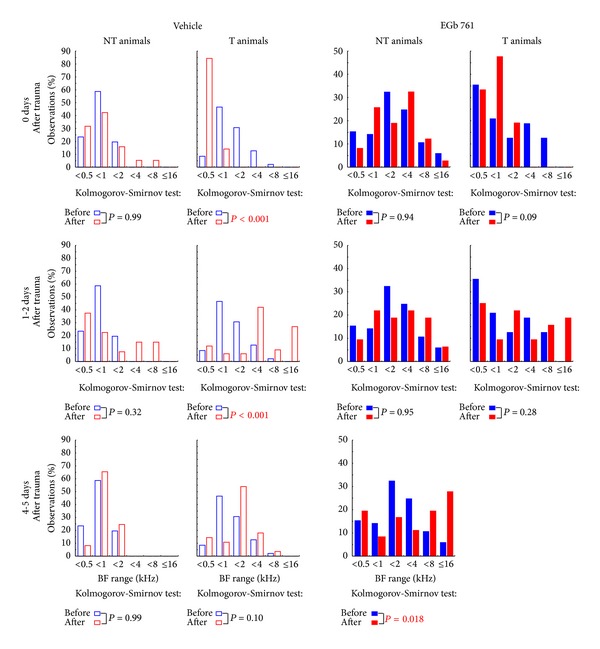
Changes in BF frequency distributions over time. Shown are the comparisons of the frequency distributions of BF (observations in %) binned in one octave step of vehicle treated animals (left two columns) and EGb 761-treated animals (right two columns). Treated and untreated animal groups are further subgrouped into NT (first and third column) and T animals (second and fourth column) before the trauma (blue) with the data obtained during 3 different time points windows after trauma (red), from top to bottom: day of trauma, 1 to 2 days after trauma, and 4 to 5 days after trauma. The distributions are tested by Kolmogorov-Smirnov tests corrected for multiple comparisons. Note that we were not able to record from the single animal in the EGb 761 tinnitus group at days 4 to 5.

In addition to these overall changes in AI activity, we also found plastic changes of the tonotopic organization in AI, as evident from changes in mean BF (Figure 8) and BF frequency distributions (Figure 9). These were different between E and V as well as T and NT animals. In NT animals we saw an effect of EGb 761 treatment already before the induction of NIHL. Treated animals showed a frequency distribution of BFs that was significantly shifted to higher frequency ranges compared to vehicle treated controls (Figure 8(a): Tukey post-hoc-test, *P* = 0.05; and Figure 9, compare blue bars in first versus third column: Kolmogorov-Smirnov-test, *P* = 0.04). In both NT groups (V-NT and E-NT), NIHL introduced no further effect on mean BF (Figure 8(a); Tukey post-hoc-tests, *P* > 0.05), but a significant flattening of the BF frequency distribution after 4 to 5 days after trauma (Figure 9, third column; Kolmogorov-Smirnov-test, *P* = 0.018). By contrast, in T animals no significant difference was found in BF distribution between the V and E groups before NIHL (Figure 8(b); Tukey post-hoc-test, *P* > 0.05; and Figure 9; compare blue bars in second versus fourth column; Kolmogorov-Smirnov-test, *P* > 0.05), pointing to a neurophysiological correlate of a possible division of EGb 761 treated animals in responders and nonresponders (blue circles in Figure 8; compare also blue curves in Figure 7(c)). In response to NIHL, V-T animals showed disturbances in tonotopic organization, as evident by significant shifts in mean BF (Figure 8(b); Tukey post-hoc-test, *P* < 0.001); and shifts in BF frequency distribution (Figure 9, second column day 0 and day 1-2; Kolmogorov-Smirnov-test, *P* < 0.001) that normalized after 4 to 5 days. In the E-T group, no such shifts were seen (Figure 8, right panel; Tukey post-hoc-test, *P* > 0.05; see also Figure 9 fourth column; Kolmogorov-Smirnov-test, *P* > 0.05). Note that in group E-T no data could be measured 4 to 5 days after trauma due to problems with the recording chamber after day 3 after trauma in the only T animal of the E group.

#### 3.2.3. Neuronal Threshold and Spectral Tuning Sharpness

**Figure 10:**
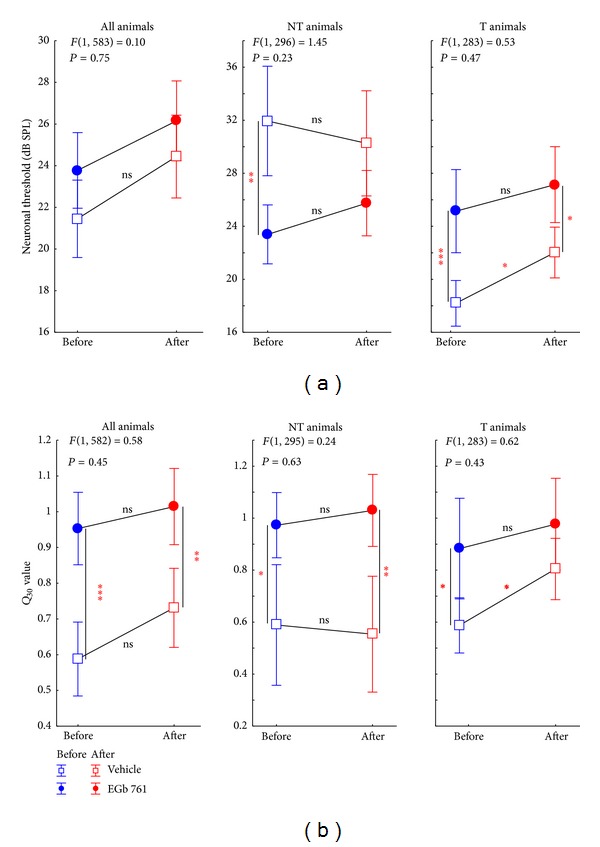
Trauma and treatment induced changes of neuronal response characteristics in NT and T animals. (a) Statistical interaction (with *F*-statistics) of time of measurement (before versus after trauma) and animal group (V versus E) with the mean neuronal threshold (±95% confidence interval) averaged across all animals (left panel) or separated into nontinnitus (center panel) and tinnitus animals (right panel). (b) Statistical interaction of time of measurement and animal group on spectral tuning sharpness (*Q*_30_ value) with the same grouping as above. Note that none of the statistical interactions become significant while most data show significant differences between V and E animals in the Tukey post-hoc-tests indicated by the asterisks (ns = not  significant, *P < 0.05, **P < 0.01, ***P < 0.001).

NIHL and EGb 761 treatment also affected neuronal thresholds and sharpness of spectral tuning measured in AI (Figure 10). When mean neuronal thresholds in AI were compared across all experimental groups, no significant differences were observed between V and E animals, neither before nor after NIHL (Figure 10(a), left panel; Tukey post-hoc-tests, all *P* > 0.05). Interestingly, when T and NT animals were analyzed separately (Figure 10(a), middle and right panel), another possible predisposition for the development of tinnitus after NIHL was seen in the V group: NT animals showed much higher neuronal thresholds in AI before NIHL compared to T animals (Student *t*-test, *P* < 0.001). These low thresholds in V-T increased after NIHL (Tukey post-hoc-test, *P* = 0.02), whereas no significant changes were observed in V-NT animals (Tukey post-hoc-test, *P* > 0.05). In the EGb 761 treated group, these pre-NIHL differences vanished, resulting in an intermediate level of neuronal thresholds that did not differ between T and NT animals (Student's *t*-test, *P* > 0.05) and remained stable even after NIHL (Tukey post-hoc-tests, *P* > 0.05).

Analysis of spectral tuning sharpness as specified by *Q*_30_ values revealed another pre-NIHL effect of EGb 761 treatment (Figure 10(b)): E animals showed significantly increased tuning sharpness across most experimental groups and conditions tested (Tukey post-hoc-tests, *P* < 0.05), with the exception of the V-T versus E-T comparison, where the Tukey post-hoc-tests showed *P* > 0.05. At least for the NT groups, where the E animals had lower neuronal thresholds compared to the controls, this result points to a neuroplastic process triggered by the EGb 761 treatment, which is effective in off-BF frequency ranges.

#### 3.2.4. Response Latency and Response Duration

**Figure 11:**
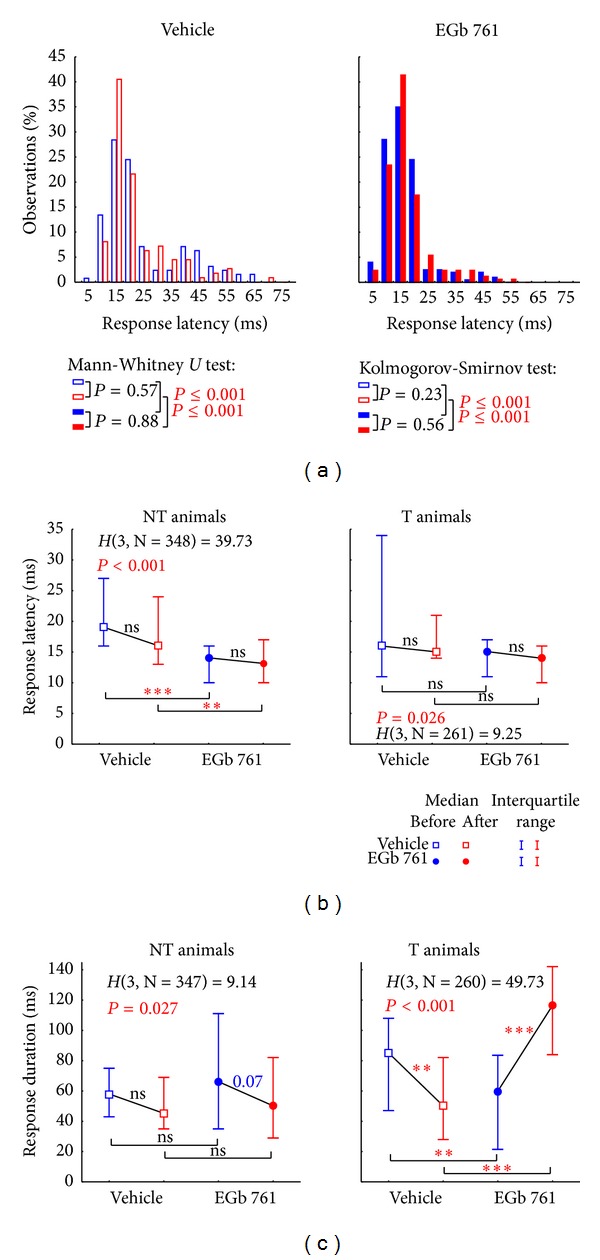
Distributions and Kruskal-Wallis-ANOVAs of neuronal response latency and duration in NT and T animals. (a) Distribution of response latencies (given in % observations binned into 5 ms bins) before (blue) and after (red) trauma in vehicle treated (open symbols) and EGb 761-treated animals (solid symbols) with the median values and interquartile range given above. Additionally, the statistics of the Mann-Whitney *U*-tests (corrected for multiple comparisons)—testing of median and interquartile range—and the Kolmogorov-Smirnov tests for the testing of the whole distributions against each other are plotted. Note that in both tests only the two pre- and two postdatasets between the groups are significantly different from each other while pre- versus posttrauma data are equal in both animal groups. (b) Median neuronal response latency (in ms ± interquartile range) tested by Kruskal-Wallis-ANOVAs (*H*-statistics) and multiple comparisons between the subgroups (ns = not  significant, **P < 0.01, ***P < 0.001) separated in nontinnitus and tinnitus perceiving animals treated with vehicle or EGb 761 before and after trauma. (c) Median neuronal response duration (in ms ± interquartile range) was analyzed as above.

The details of our analyses of temporal neuronal response properties to tones in AI are shown in Figure 11. Here Figure 11(a) gives an overview of the frequency distribution of response latencies measured in V animals (open bars) and E animals (filled bars) both before NIHL (blue) and after NIHL induction (red) summarized across all recording sessions from 0 to 5 days after trauma. As revealed by both the Kolmogorov-Smirnov test for the comparison of distributions and the Mann-Whitney *U*-tests for the comparisons of the median values (see insets) there were no differences in pre- versus post-NIHL latency distributions in either V or E animals. Nevertheless, E animals had significantly shorter latencies than V animals under both conditions. When analyzing NT and T animals separately (Figure 11(b), Kruskal-Wallis ANOVA with post-hoc Median-tests), the difference in mean latency in NT animals was 5 ms before and 3 ms after NIHL. Such differences were not seen in T animals, pointing to another possible distinction of EGb 761 responders and nonresponders. In contrast, when comparing response duration (Figure 11(c)), there were no significant differences between pre- and post-NIHL conditions, neither in the V-NT nor in the E-NT group, although in the latter there may be a trend to shorter response durations after the trauma (Figure 11(c), left panel). In the T groups on the other hand, NIHL induced a significant shortening in response duration in controls while it induced significant increase in response duration after EGb 761 treatment.

### 3.3. Neurophysiological Effects of Prophylactic EGb 761 Treatment: Comparison between Rate-Intensity Functions in Brainstem and Auditory Cortex

**Figure 12:**
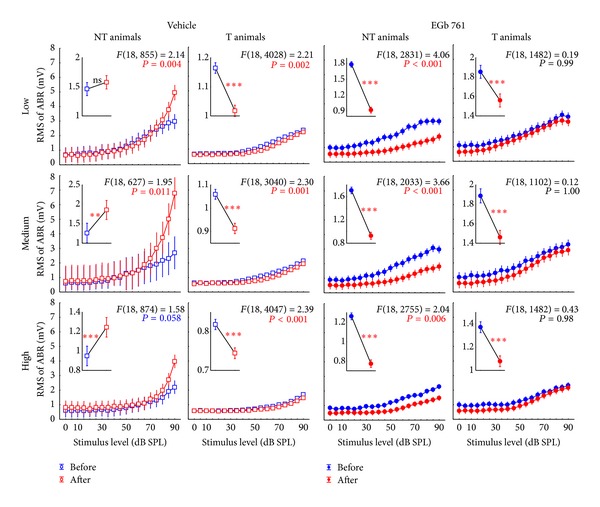
Level functions of the auditory brainstem responses (ABR) in NT and T animals grouped for vehicle and EGb 761 treated groups. Given are the mean root mean square (RMS) values of the ABR amplitudes (±95% confidence intervals) as a function of stimulus level before (blue) and after trauma (red) for the four subgroups for low (0.5 to 1.4 kHz), medium (2.0 to 4.0 kHz), and high stimulation frequencies (5.6 to 16.0 kHz). The *F*-statistics of the 2-factorial ANOVAs are shown for each panel and the corresponding 1-factorial part grouped for time of measurement (pre versus post trauma) is given in each inset (also with the RMS of ABR in mV) with the asterisks indicating the significance level (ns = not  significant, **P < 0.01, ***P < 0.001).

**Figure 13:**
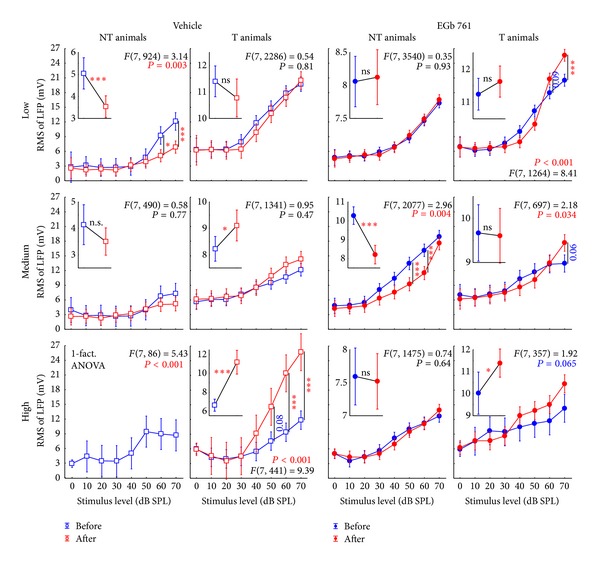
Level functions of the local field potential (LFP) amplitudes in the auditory cortex of NT and T animals grouped into vehicle and EGb 761 treated groups. Presented are the mean RMS values of the LFP amplitudes (±95% confidence intervals) as a function of stimulus level grouped as in Figure 8.

**Figure 14:**
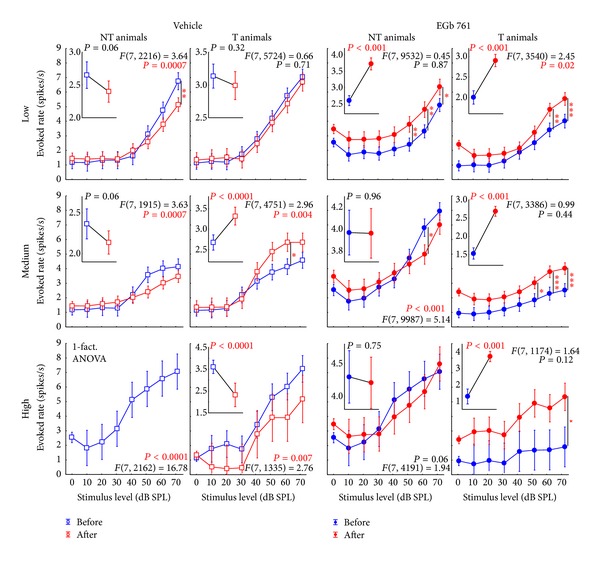
Level functions of the evoked spike rates in the auditory cortex of NT and T animals grouped into vehicle and EGb 761 treated groups. The mean evoked response rates of the auditory neurons in AI (±95% confidence interval) as a function of stimulus level grouped as in Figure 8 are shown.

**Figure 15:**
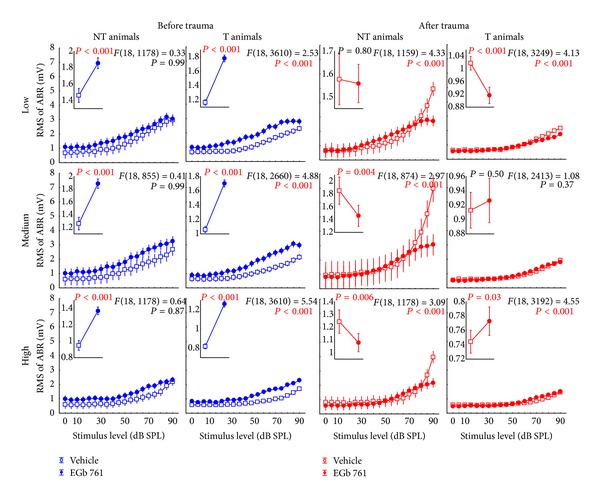
Replotted data of Figure 8, grouped according to the time of measurement (before versus after trauma) to allow for an easier comparison of vehicle versus EGB 761 treated animals. Note the consistent differences in the ABR amplitudes, especially for pretrauma in vehicle versus EGB 761 treated animals.

**Figure 16:**
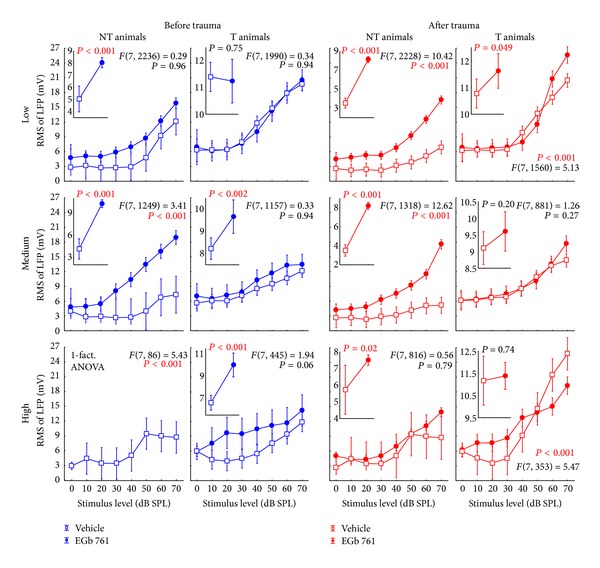
Replotted data of Figure 9, grouped according to the time of measurement (before versus after trauma) to allow for an easier comparison of vehicle versus EGB 761 treated animals. Note the consistent differences in the LFP amplitudes, in particular in the NT animals in vehicle versus EGB 761 treated groups.

**Figure 17:**
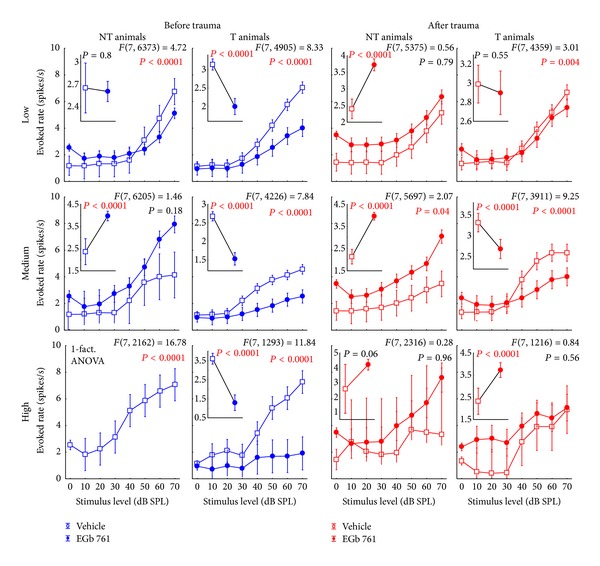
Replotted data of Figure 10, grouped according to the time of measurement (before versus after trauma) to allow for an easier comparison of vehicle versus EGB 761 treated animals.

**Figure 18:**
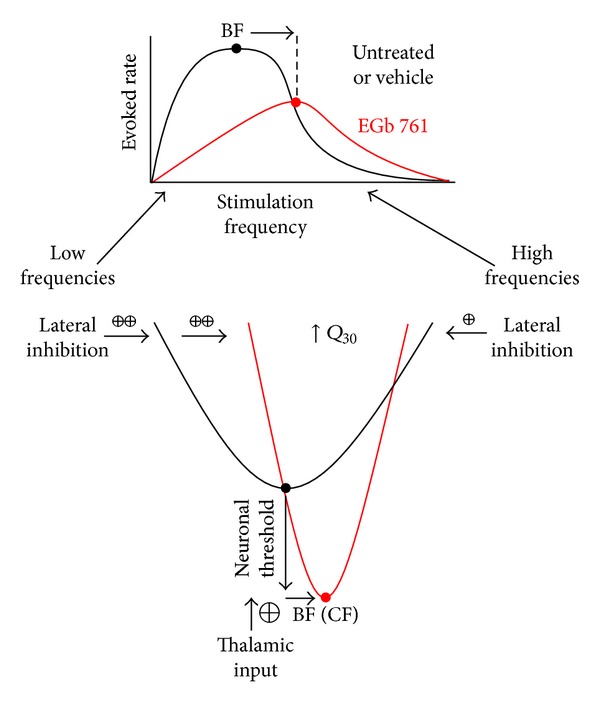
Proposed model of the effects of EGb 761 treatment on auditory processing. Upper panel: evoked neuronal response rate in AI for iso-intensity pure tone stimuli in vehicle (black) and EGb 761 treated animals (red). Lower panel: neuronal threshold and tuning of cortical neurons in both animal groups. Based on our data, we propose two main effects of EGb 761 on auditory processing: first, an increase of auditory brainstem activity leading to an increased thalamic input to AI, which results in lower response thresholds and shorter response latencies, and second, an asymmetric effect on lateral inhibition in AI that reduces overall response rates, shifts the best frequency (BF) to higher values, and sharpens spectral tuning (*Q*_30_-values).

Finally, we compared the mean rate-intensity functions based on tone evoked ABR as a measure of brainstem activity (Figure 12); local field potentials (LFP) in AI, as an estimate of AI input (Figure 13; although LFP reflect thalamic as well as intracortical input [43]); and neuronal spike counts in AI, as a measure of AI output (Figure 14). In each of these figures, data are given for V (left) and E groups (right), and each of these separately for NT (first and third columns) and T animals (second and fourth columns). Furthermore, data are grouped for responses to three ranges of stimulation frequencies, namely, low (upper row, 0.5 to 1.4 kHz for ABR and 0.25 to 1.4 kHz for LFP and spiking activity), medium (middle row, 2.0 to 4.0 kHz), and high (lower row, 5.6 to 16.0 kHz) frequency tones. In each single panel, the effect of NIHL on the neuronal activity can be estimated by comparing pretrauma conditions (blue) with the posttrauma status (red). A 2-factorial ANOVA is used for this comparison. In addition to this comparison of pre- versus post-NIHL neuronal activity shown in Figures 12 to 14, the same data are replotted in Figures 15 to 18, respectively, to allow for an easier comparison of neuronal activity in groups V (open symbols) versus E (filled symbols). That is, Figures 12 to 14 show the effects of NIHL on neuronal activity throughout the auditory system; Figures 15 to 18 show the effects of prophylactic EGb 761 treatment on this activity. Insets in each panel in these figures give mean values across the respective rate-intensity function (as 1-factorial part of the 2-factorial ANOVA).

In general, both NIHL and prophylactic EGb 761 treatment led to significant changes in rate-intensity function at all levels of the auditory pathway (brainstem, AI presynaptic, AI postsynaptic), and within all frequency ranges analyzed here. These changes may be evident in absolute shifts of the function (significant change in mean values in the 2-factorial ANOVA), different shapes of the function (significant interaction in the 2-factorial ANOVA), or both. For better readability the *F* and *P* values of all tests are only shown in the appropriate figures and not mentioned again in the text.

Evaluating the changes at the level of the auditory brainstem (Figures 12 and 15), a general decrease in neuronal activity after NIHL was evident in all groups except for the group V-NT (Figure 12, first column), where a slight increase in neuronal activity was observed that was manifest especially at high stimulus intensities. EGb 761 treatment led to a slight increase in auditory brainstem activity before NIHL in all groups and frequency ranges (Figure 15, compare blue functions on the right to the blue functions on the left). NIHL led to a decrease in brainstem activity in all E animals (Figure 12, right), but the decrease was stronger in animals that did not develop tinnitus (group E-NT, Figure 12, third column), which is clearly different from the V-NT ABR described above. In other words, whereas the ABR in T animals reacted similarly both in EGb 761 and vehicle treated animals, NT animals in groups V and E obviously deployed different neuroplastic mechanisms in the auditory brainstem that prevent the development of tinnitus after NIHL.

When looking to the synaptic input into AI further upstream in the auditory pathway (Figures 13 and 16), the picture becomes less clear compared to the auditory brainstem. For the control group V, effects of NIHL in general seem to point into the opposite direction as we just described for the ABR measurements: where we saw increases in activity after NIHL in the ABRs (Figure 12, NT animals, first column) we now find decreased LFP activity (Figure 13, first column) and vice versa (Figures 12 and 13, T animals, second columns). As in the brainstem activity, LFP changes were again mainly restricted to high stimulus intensities. In the EGb 761 treated animals, in contrast to the ABR results, NIHL-induced changes were much more specific and restricted to small ranges of frequency and intensity. In E-NT animals, for example, (Figure 13, third column), significant decreases in activity were exclusively seen for medium frequencies at 50 to 60 dB SPL, pointing to a very specific mechanism focused on the traumatized frequency range to prevent tinnitus development. Interestingly, a change in activity was already obvious before the induction of NIHL (Figure 16, first column). Prophylactic treatment with EGb 761 led to strong increases in LFP activity before noise trauma, possibly enabling the system to react to NIHL with LFP decreases focused to the traumatized frequency range to prevent tinnitus development. Changes in the E-T group (i.e., animals that were not able to prevent the development of tinnitus) after NIHL were much less focused and showed increases in activity rather than decreases (Figure 13, fourth column). Furthermore, in this group, we did not observe increases in LFP activity before noise trauma, which differ from the E-NT group (Figure 16, second column), at least in low and medium frequency ranges, pointing to another possible distinction between EGb 761 responders and nonresponders.

Finally, analyzing AI output activity (Figures 14 and 17), we generally found similar effects of NIHL in the V group, except for high stimulation frequencies in T animals. There, the strong increase that was evident in the LFP data (Figure 13, second column, bottom panel) turned into a general decrease in spiking output functions (Figure 14, second column, bottom panel). In the E group, the picture was again similar in AI output compared to AI input functions in NT animals, except for an increase in AI responses after NIHL to low stimulus frequencies (cf. Figure 14, third column versus Figure 13, third column). E-T animals on the other hand showed strong differences in the NIHL-induced changes in AI input versus output functions, with a general increase in AI spiking responses in all stimulation frequency ranges that was not evident in LFP functions and may be a correlate of tinnitus. Note that the increase in spiking activity increased from low to high stimulation frequencies; that is, it was particularly strong at frequencies corresponding to the behaviorally determined perceived tinnitus frequencies [31]. Evaluating the effect of EGb 761 treatment on spiking activity in AI (Figure 17), spike rates tended to be increased for most frequency and intensity ranges in the E-NT compared to V-NT animals, both before and after NIHL. By contrast, in E-T animals, we generally saw decreases in evoked spike rates in AI before NIHL compared to V-T animals. After NIHL, differences between E-T and V-T animals differed as a function of stimulation frequency, with least differences seen at low stimulation frequencies and strongest differences at high stimulation frequencies.

### 3.4. Known Physiological Effects of EGb 761

EGb 761 is a standardized extract of dried green leaves of* Ginkgo biloba*. It contains numerous different compounds (see Section 2 and [17, 19, 44]). A number of different physiological effects are described for the EGb 761 [19, 20], but given the nature of such plant extracts as being composed of a variety of different components, it is not always easy (if at all possible) to attribute a particular effect to a single compound (although in some cases at least the effective compound class could be determined, [26, 28, 45]). In the context of the present study, four physiological effects described for EGb 761 seem to be most important for the prophylactic effects on NIHL and tinnitus development reported here. These are, first, stabilization of mitochondrial respiratory chain metabolism and ATP production due to antioxidant effects that reduce oxidative stress by elimination of ROS [17, 18]; second, increase of extracellular dopamine levels in prefrontal cortex that may improve mood and thereby reduce stress [26], based on blocking dopamine reuptake via the norepinephrine transporter [28]; third, reduction of hormonal stress responses by reduction of corticotropin-releasing hormone (CRH), adrenocorticotropic hormone (ACTH), or corticosterone [46]; and fourth, improved blood flow [47, 48].

In the final section we will discuss how these known physiological effects of EGb 761 may be beneficial in the context of a reduction of NIHL and central tinnitus development.

### 3.5. Possible Mechanisms of EGb 761 Protective Effects against NIHL and Noise Trauma-Induced Tinnitus

A number of studies have demonstrated that the prophylactic use of several antioxidant substances may reduce NIHL [14, 49]. Models for NIHL assume that the cochlea is damaged mechanically by intensive noise, and in addition that metabolic stress induces hair cell death via ROS, which activate apoptotic pathways. According to these pathological mechanisms antioxidant substances are thought to protect the cochlea from hair cell loss after intensive noise exposure by reducing ROS. Over the last few years it has been demonstrated numerous times that this strategy can successfully be applied using different antioxidants [50–58]. It is therefore plausible to assume that the antioxidant effect described for EGb 761 is responsible for the protective effect of the extract against NIHL as reported here (Figure 3) and earlier [15, 16]. In addition, as noise trauma also decreases cochlear blood flow [59], the improvement of blood flow after EGb 761 administration [19] may also add to the cochlea protective effect of the extract.

As a consequence, it seems self-evident that a reduced amount of NIHL will also lead to a reduced percentage of animals that develop noise trauma-induced tinnitus (Figure 5). In this context it remains unclear if the tinnitus we observed in our animal model in this study does reflect acute tinnitus or already a chronic manifestation of tinnitus. But independent of the type of tinnitus, we observed only in T animals an isolated increase in gap response amplitude (cf. Figure 5(a)) after the acoustic trauma, while NT animals showed a generally similar pre- and posttrauma gap response. We know from our earlier study [31] that central neuroplastic changes in AC which correlate with the development of tinnitus in our animal model are restricted to the first week after trauma, although the tinnitus percept itself is stable for at least 16 weeks. It may therefore be the case that in this model system chronic tinnitus already manifests after one week after trauma. However, this is still an open question that needs to be addressed in future studies. Independent of this question, we were able to describe a number of additional effects of the extract on central auditory processing, which make it unlikely that all beneficial effects we observed on NIHL and tinnitus development may be based exclusively on the abovementioned protective effects on the peripheral auditory system, that is, on hair cells.

In a previous publication on tinnitus development in untreated animals [31] we proposed a global inhibitory mechanism in AI that should be able to successfully counteract the development of tinnitus in a subset of animals by decreasing overall activity in AI. In this report, we found that administration of Ginkgo extract before the trauma already leads to a reduction in AI activity, which is comparable to that observed in untreated animals after trauma (cf.  Figure 7). Nevertheless, closer inspection of the data showed that the pretrauma effects of EGb 761 on activity in the central auditory system do not resemble the posttrauma mechanism that prevents some of the untreated animals from the development of tinnitus. Importantly, we saw an increase in activity in auditory brainstem before the trauma in EGb 761 treated animals, whereas there was a slight decrease of ABR amplitudes in untreated animals after trauma [31] (cf. Figure 14). Furthermore, EGb 761 treatment led to increased mean BFs, which indicate plastic changes in tonotopic organization in AI that were not seen in untreated animals without tinnitus.

Based on these differences we propose here the hypothesis that EGb 761 treatment activates a lateral inhibition mechanism rather than a global inhibitory mechanism as proposed for untreated animals.  Figure 18 illustrates the details of this model. The model assumes two main effects of the Ginkgo extract on central auditory processing, namely, an increase of gain in auditory brainstem, as evident from increased ABR amplitudes (Figure 15), and an activation of intracortical lateral inhibition, as indicated by increased tuning sharpness (Table 1, *Q*_30_). We believe that the increased ABR activity leads to an increased thalamic input to AI, which is responsible for the decreased neuronal thresholds we observed after Ginkgo treatment (Table 1, top row; Figure 18). The reduced response latency after EGb 761 treatment is probably a consequence of these reduced thresholds. Nevertheless, as the evoked response rate is decreased in AI, some intracortical inhibition must be activated simultaneously. The increased *Q*_30_ values point to local, lateral inhibitory influences, while the shifted mean BF points to an asymmetric distribution of this lateral inhibition, with stronger inhibition at the low frequency side compared to the high frequency side of a given tuning curve (Figure 14, bottom panel). The fact that the main reduction in overall activity in AI is in the low frequency rather than in the high frequency range (Figure 7, left column; Figure 18, top panel) is in line with this interpretation. As a result, EGb 761 induced changes in activity throughout the auditory system seem to lead to processing characteristics in AI that are more stable (Figures 7(c), 8 and 10) and therefore less prone to the development of central tinnitus after noise trauma (Figure 5). The fact that most of these effects of EGb 761 treatment on AI activity were not seen in animals that did develop tinnitus and that most pretrauma changes remained stable in the NT but not in T animals (Table 1, third row) leads us to speculate that these central effects of EGb 761 in responders compared to nonresponders substantially add to the protective effect of the antioxidant characteristics in the cochlea that counteract NIHL, but revealing the exact mechanism needs further investigation.

Finally, one could speculate about these mechanisms by which EGb 761 leads to the changes in central auditory processing as described above. One factor in this context may be the increased dopamine level in prefrontal cortex that was found under EGb 761 treatment [26]. Dopamine is known to foster several neuroplastic processes [60–62] so that it may be possible that dopamine effects are also involved in the neuroplasticity described here under treatment, although the mechanisms that trigger this plasticity still remain unclear. In addition, dopamine is known to improve mood, and when combined with the EGb 761 effects on hormonal stress responses, these factors may lead to decreased stress in the animals that could also be beneficial in the context of tinnitus development [63].

As described above, the exact mechanism that leads to the increased lateral inhibition in AI after the treatment with EGb 761 remains unclear. But the concept that such lateral inhibition may counteract the development of central tinnitus—especially in the acute phase after a noise trauma—seems to be straightforward based on current models of central tinnitus [64] and was already used in new, promising treatment strategies in both animal models for tinnitus [65] and human patients [66]. Possibly, additional administration of EGb 761 might further improve the outcome of such treatment regimens.

## 4. Conclusion

In this report we were able to demonstrate that the prophylactic treatment of animals with the* Ginkgo biloba* extract EGb 761 elicits a number of protective effects on the development of NIHL as well as on subjective tinnitus, both on peripheral as well as central levels of the auditory pathway. Although the fact that only a subset of animals that have been characterized behaviorally could also undergo detailed electrophysiological recordings might pose a limitation to this study (EGb 761 animals: 3 NT, 1 T; vehicle animals: 1 NT, 2 T), the observed effects of EGb 761 on central auditory processing still revealed a number of significant changes of response parameters that allow us to speculate about the neurophysiological mechanisms underlying these changes. A qualitative overview of these effects is given in Table 1.

In general, when comparing EGb 761 effects between NT and T animals (upper two rows versus lower two rows in Table 1) it is obvious that in NT animals much more significant effects of the extract can be seen than in T animals. Furthermore, where effects were found in T animals, they sometimes pointed into the opposite direction as in NT animals (Table 1, threshold), or there were no effects in NT animals (e.g., Table 1, response duration). Based on these differences, in particular those that were already seen before trauma, animals can be separated in EGb 761 responders and nonresponders. This distinction seems to correlate with the distinction between NT and T animals, respectively. That is, the extract, if prophylactically applied, obviously is able to reduce NIHL (Figure 3) and the probability to develop subjective tinnitus after noise trauma (Figure 5), and this outcome is based on a whole number of neurophysiological effects (Figures 7 to 17).

## Figures and Tables

**Table 1:** Overview of parameter change EGb 761 versus vehicle control.

	Hearing threshold	Evoked rate	Mean BF	BF distrib.	Neuronal threshold	*Q*_30_	Response latency	Response duration
Nontinnitus animals								
Before	—	↓	↑	↑	↓	↑	↓	—
After	↓ (acute & 1–7 d)	—	↑	↑	—	↑	↓	—
Tinnitus animals								
Before	—	—	—	—	↑	↑	—	↓
After	— (1 & 7 d)	—	—	—	↑	—	—	↑
